# Preventive Measures For pressure injuries: structure of social representations of nursing teams

**DOI:** 10.1590/1980-220X-REEUSP-2022-0012en

**Published:** 2022-10-24

**Authors:** Rosa Maria Ferreira de Almeida, Luiz Fernando Rangel Tura, Rafael Celestino da Silva

**Affiliations:** 1 Universidade Federal do Rio de Janeiro, Escola de Enfermagem Anna Nery, Rio de Janeiro, RJ, Brazil.; 2 Universidade Federal do Rio de Janeiro, Instituto de Estudos e Saúde Coletiva, Rio de Janeiro, RJ, Brazil.; 3 Universidade Federal do Rio de Janeiro, Escola de Enfermagem Anna Nery, Departamento de Enfermagem Fundamental, Rio de Janeiro, RJ, Brazil.

**Keywords:** Pressure Ulcer, Patient Safety, Nursing, Psychology, Social, Úlcera por Presión, Seguridad del Paciente, Enfermería, Psicología Social, Lesão por Pressão, Segurança do Paciente, Enfermagem, Psicologia Social

## Abstract

**Objective:**

To analyze the structure of social representations on the preventive measures to pressure injury of nursing teams.

**Method:**

Qualitative research, based on the structural approach of Social Representations. It was conducted with 103 nursing professionals from a specialized hospital, using the Word Association Test. Data were submitted to prototypical analysis, using two analysis techniques of centrality, similarity and double negation.

**Results:**

The evocations “care” and “decubitus” made up the central core of the representation, which was structured based on the imagery dimension of changing position, from which a hierarchy of preventive measures occurs. The term “care” referred to the normative dimension of representation as a responsibility of nursing, and to the practical dimension, as preventive actions with the use of technologies.

**Conclusion:**

The image, normative and practice dimensions are part of the representation structure and guide compliance with preventive measures.

## RESUMO

**Objetivo:**

Analisar a estrutura das representações sociais sobre as medidas preventivas à lesão por pressão da equipe de enfermagem.

**Método:**

Pesquisa qualitativa, pautada na abordagem estrutural das Representações Sociais. Foi realizada com 103 profissionais de enfermagem de um hospital especializado utilizando-se a Técnica de Associação Livre de Palavras. Os dados foram submetidos à análise prototípica, e empregou-se duas técnicas de análise da centralidade, de similitude e da dupla negação.

**Resultados:**

As evocações “cuidado” e “decúbito” compuseram o núcleo central da representação, que se estruturou com base na dimensão imagética da mudança do decúbito, a partir da qual ocorre uma hierarquização das medidas preventivas. O termo “cuidado” remeteu à dimensão normativa da representação como uma responsabilidade da enfermagem, e à dimensão prática, pelas ações preventivas com o emprego de tecnologias.

**Conclusão:**

As dimensões da imagem, normativa e da prática integram a estrutura da representação e orientam a adesão às medidas preventivas.

## RESUMEN

**Objetivo:**

Analizar la estructura de las representaciones sociales sobre las medidas preventivas contra las lesiones por presión del equipo de enfermería.

**Método:**

Investigación cualitativa, basada en el enfoque estructural de las Representaciones Sociales. Se realizó con 103 profesionales de enfermería de un hospital especializado, utilizando la Técnica de Asociación de Palabras Libres. Los datos fueron sometidos a análisis prototípico, utilizando dos técnicas de análisis de centralidad, similitud y doble negación.

**Resultados:**

Las evocaciones “cuidado” y “decúbito” conformaron el núcleo central de la representación, la cual se estructuró a partir de la dimensión imaginaria del cambio de posición, a partir de la cual se da una jerarquía de medidas preventivas. El término “cuidado” se refería a la dimensión normativa de representación como responsabilidad de enfermería, ya la dimensión práctica, como acciones preventivas con el uso de tecnologías.

**Conclusión:**

Las dimensiones imagen, normativa y práctica forman parte de la estructura de representación y orientan la adhesión a las medidas preventivas.

## INTRODUCTION

Pressure injury (PI) is a localized damage to the underlying skin and/or soft tissues, usually on a bony prominence, and may be related to the use of a medical device or to another artifact^(1)^. It is one of the most common consequences of prolonged hospitalization, most prevalent when it combines with the presence of risk factors such as advanced age, bed restriction, chronicity of pathology, among others^(2)^.

From a clinical and economic point of view, PI are responsible for high rates of complications and mortality, representing costly expenses for health systems. Such clinical and financial impacts are added to physical and emotional distress, which can lead to a further worsening of patients’ general condition and an increase in their dependence, prolonging treatment and cure^(3,4)^.

From an epidemiological point of view, PI have high incidence rates^(5,6)^. A systematic review quantified the prevalence, incidence, and rate of hospital-acquired PI by adult patients and identified the most frequently occurring stages and the most affected anatomical location. Forty-two studies were included, with a total sample of 2,579,049 patients. The combined prevalence of 1,366,848 patients was 12.8%; the combined incidence of 681,885 patients was 5.4 per 10,000 patient-days; and the acquired PI rate of 1,893,593 patients was 8.4%. The most frequent stages were I (43.5%) and II (28.0%), and the most affected sites were the sacrum, heels and hip^(5)^.

In the particularity of a hospital in northern Brazil, the context of this research, in the data notified to the Brazilian National Health Regulatory Agency (*Agência Nacional de Vigilância Sanitária*), between July 2019 and June 2020, among the notifiable security incidents, PI was the most frequent event, with emphasis on 17 stage III PI and one death.

This evidenced problem of PI brings a reflection on the intrinsic and extrinsic risk factors to patients that are subject to modification, with a view to PI prevention. In this sense, preventive measures to PI stand out, whose adoption is the responsibility of managers and care professionals who make up the health system multidisciplinary teams. Among the main PI preventive measures are: daily assessment of skin integrity using specific scales; protection of patient skin from excess moisture, dryness, friction and shear; nutritional status management; maintenance of skin hydration; pressure redistribution through decubitus change; encouraging early mobilization; and use of appropriate pressure relief devices, among others^(1,7)^.

The team’s compliance with preventive measures is essential to reduce the risk of PI occurrence, in particular, of nursing teams, since these professionals promote continuous and direct assistance to patients. Studies involving nursing professionals indicate that preventive measures may not be incorporated into care practice, as in fact they should be^(8–11)^.

An example of this is the research in Mexico, which identified the nursing care omitted for PI prevention from nursing team perception and the assessment of patients at risk or with PI based on observation. The mean care omitted score in the team’s perception was 29.95, on a scale of 0 to 100 points. The highest rates of omission were for skin care (38.5%), registration of predisposing factors to PI (33.5%) and patient repositioning every two hours (31.1%)^(12)^.

In the assessment of the care to be performed, the score for care omitted was 52.01%, with omissions predominating, use of pressure relief in bony prominences (58.6%), patient positioning with good body alignment (58.2%), use of air mattresses (57.6%) and patient repositioning every two hours (54.5%)^(12)^.

A survey of 1,806 nurses from 10 hospitals in China analyzed the attitude of nurses towards PI prevention. The attitude scale ranged from 13 to 52 points, and 53.4% had a positive attitude. The mean attitude score was 40.80. The statement “I have an important task in prevention” obtained the agreement of 99% of participants, and 30% (540) disagreed with the statement “A lot of attention goes to PI prevention”^(10)^.

Considering this production of knowledge on the subject and the researcher’s care observations, which brought indications of failures in compliance of nursing professionals with preventive measures, the assumption was raised that the application of measures to prevent PI in hospitalized patient care practices was related to the existence of social representations (SR) about preventive measures by nursing professionals.

SR objects are psychosociological, i.e., they are part of the subjects’ subjective and social dimensions. These, in turn, integrate the information from the scientific universe with that which circulates in the social environment, added to the knowledge originating from their own experiences, thus re-elaborating the knowledge, which guides their positions and conducts^(13)^. SR have a polymorphic composition that includes concepts, propositions and explanations originated in the interpersonal communications of everyday life, woven by a set of elements of a diverse nature, such as cognitive processes, social insertions, affective factors and value system^(13)^.

In the case of PI prevention measure, it is considered that it is a salient phenomenon^(14)^, i.e., that mobilizes the affective, valuative and symbolic dimensions, which are part of SR elaboration^(13)^. Affections can come from the physical effort of nursing professionals to perform these care activities, in addition to the involvement with the eliminations and odors of patients’ body. As for values, the implementation of some of the preventive measures is delegated, in many moments, to professionals of technical level; therefore, due to the lower level of qualification, it can refer to a valuation of this care. The symbolic dimension may be related to the classification of prevention as a lower priority activity, based on the hierarchization of care activities to be carried out.

From the above, it is understood that the adoption of preventive measures does not depend only on information or the existence of inputs, but also on subjective elements that form a network of meanings about this phenomenon. Therefore, understanding the nursing team’s SR can contribute to a critical reflection on attitudes and conducts adopted in care practice, allowing, from this, the adoption of strategies to sensitize these professionals with regard to such behaviors.

The objective was to analyze the SR structure on PI preventive measures of nursing teams.

## METHOD

### Study Design

This is qualitative research, having the SR theory as framework^(13)^. SR, due to its practical character, guide the action of individuals in the world, with emphasis on social relationships. Thus, its application helps to identify and understand the motivations of nursing professionals and the subjective elements that interfere in the practices adopted regarding PI preventive measures, object of this investigation. The SR structural approach was applied to identify the SR core, its organization and constitutive elements^(13,14)^.

### Search Setting

The study was carried out in a tertiary-level state hospital located in the city of Porto Velho, Rondônia, Brazil, with 100 active beds. It is an institution linked to the Unified Health System (*Sistema Único de Saúde*), a reference in the state and in the North region for the treatment of infectious diseases. Its monthly average is 20,000 visits and it has three medical clinic units, an isolation sector and the Intensive Care Unit (ICU) as inpatient clinics, sectors that constituted the locus of research, developed from October to December 2019.

Preventive measures are established by the Patient Safety Center through the Standard Operating Procedure of PI preventive measures, which was built with the nursing team in 2019. The institution has a wound treatment committee, composed of a nurse and a nursing technician.

### Participants and Selection Criteria

Participants were the nursing team members of the sectors that served as the locus. The research universe consisted of 112 potential participants, and, for sample definition, the theoretical recommendation of recruiting at least 100 participants was considered for SR structure analysis^(15)^. Based on this recommendation, recruitment included members of nursing teams, from both shifts, over 18 years old, with more than six months in the institution, time considered sufficient for the incorporation of care protocols, performing direct patient care activities. We excluded professionals who perform activities in the Emergency Room, as patients stay in this unit for a period of less than 24 hours.

For recruitment, there was an approach to the research field and sectors through contact with service managers. From this, the researcher was introduced to the sectors and the nursing team was invited, which resulted in the recruitment of 103 participants.

### Data Collection

For data collection, the Word Association Test (WAT) was applied, which is a technique that seeks to recruit the most spontaneous dimension of the subject in relation to the phenomenon^(14)^. This choice considered that the apprehension of some elements about preventive measures for PI could be masked by other production techniques.

Data collection took place between October and November 2019. It consisted of asking participants to record on a form the first five words that came to mind when they heard the expression “PI preventive measures”. Then, participants were asked to list the words evoked from one to five, from most important to least important.

The WAT was applied to the day and night shift nursing teams of all shifts in the sectors chosen for the research. At an appropriate shift time, participants were recruited at the nursing station, and a brief explanation of the research was given, followed by training in the technique application using a fictitious inducing term. Each participant was asked to say five words that came to mind after the test stimulus word. After the clarification of doubts, each participant received a clipboard with the evocation form for the registration of terms and the sociodemographic questionnaire about the professional profile regarding the sociodemographic characteristics (education, sex, length of service, training, knowledge of PI guidelines, among others).

In the second stage of data collection, the double negation centrality test (*Mise en Cause*) was applied, which is fundamental for studies of SR structural approach. The test is based on the assumption that the central elements of SR are non-negotiable and its placing in check (double negation) should necessarily induce a change in it, and the term can no longer be recognized as central by the group^(14,15)^.

The technique consisted of asking 40 participants in this study to answer the following question: can I think about PI prevention without thinking about it? (element considered central in the analysis performed with the data from the first stage). Three possible answers were proposed: 1 – Yes, I can; 2 – No, I cannot; 3 – I cannot tell.

For the technique operationalization, it was returned to the field in December 2019. Among the professionals who participated in the first stage, 40 professionals were selected to carry out this second stage. This number was defined based on a classic SR survey in Brazil, using this technique, whose phenomenon was science^(14)^. Selection occurred for convenience in the medical clinic and isolation sectors, based on the availability of professionals on the days of data collection, until reaching the established number. Participants were approached in the sectors and, those who accepted, answered an instrument with the elaborated questions.

### Data Analysis and Treatment

Data from the first stage of collection were organized with the creation of spreadsheets, which made up the research database. The first worksheet was the group profile characterization variables. Subsequently, there was the descriptive statistical treatment of these variables, with the performance of simple and percentage frequency. The second worksheet was the evocations, in which the lines contained the terms evoked by each participant. Their identification was made by a code represented by participants’ numeral order of recruitment and age.

Then, the corpus was prepared in two stages. The first standardized the words contained in the database, excluding articles and prepositions as well as adjusting the verb tenses. In the second stage, the semantic approximation was performed, simplifying and grouping synonymous or similar expressions. After this homogenization of the corpus, the final quantity of words was reached, with 516 terms, of which 47 were different.

Data were processed in the *Ensemble de Programmes Permettant L’Analyse dês Évocations* (EVOC), version 2000, from which the SR structure prototypical analysis was carried out. The prototypical analysis assumes that the SR structure’s important elements are more prototypical, i.e., more accessible to consciousness.

In this analysis, the individual (order of evocation) and collective (frequency of evocation) dimensions were considered. From this, the software generated a list of all the words evoked and proceeded to analyze the frequency and position in which they were cited. Subsequently, the mean of the Average Order of Evocation and the evocation frequency were calculated. At the end of these procedures, the four-quadrant model^(14,16)^was generated.

In this quadrant, words with high frequency and low average orders of evocation made up the upper left quadrant (ULQ) of the central nucleus. In the lower left quadrant (LLQ), contrast cognems were found, important for the subjects, but with low frequency. In the upper right (URQ) and lower right (LRQ) quadrants, the peripheral elements were allocated, with the URQ, the first periphery, with the most important elements, and the LRQ, the second periphery, with the least important elements^(16)^.

The data from the evocations passed the centrality test through the analysis of similarity, through its processing in Simi, version 2.1. The similarity analysis makes it possible to identify co-occurrence between words, therefore, the signs of connectivity between them, which reveal the structural organization of the researched group’s SR^(14,15)^.

The software analyzed the number of co-occurrences between the terms in relation to the number of participants, and, based on this similarity index, produced a schematic representation, the tree of similitude, in which the branches translate the distances, and the points are the representational elements. Thus, the central elements have connectivity with a greater number of elements and have a star shape. The next few elements can be considered peripheral^(14,15)^.

As for the results of the double negation technique, data were submitted to treatment in Excel, with calculation of the simple frequency of the three possible responses for each central term evoked. The elements that obtained a proportion of ≥75% of responses “No, I cannot”, double negation, contained in a 95% Confidence Interval, had their centrality confirmed.

### Ethical Aspects

The research was approved by the Research Ethics Committee of the researched institution, in 2018, under Opinion 4,323,665, in accordance with Resolution 466/2012. All participants signed the Informed Consent Form, ensuring their anonymity by numerical data processing.

## RESULTS

Participant characterization had a predominance of nursing technicians, with 72.81%, most of them women (87.37%), with a mean age of 43 years. Regarding the training time, the data showed an experienced group, since the highest frequency was of professionals who had between 11 and 20 years of training (54.36%). Job tenure in the hospital was diversified, with a predominance among those who had between 05 and 10 years (33%) and with less than five years (29.12%). Regarding workplace, 66 (64.05%) employees worked in the ward and 37 (35.92%) in the ICU.

Of the 28 nurses, 25 (89.28%) had a graduate degree, two of them at the master’s level and the others in the *lato sensu* modality. Of the 75 nursing technicians, 51 (68%) already had higher education. Among the participants, 56 (54%) said they did not have access to courses on PI and 81 (79%) did not have access to current guidelines for PI prevention.

**Chart 1.**
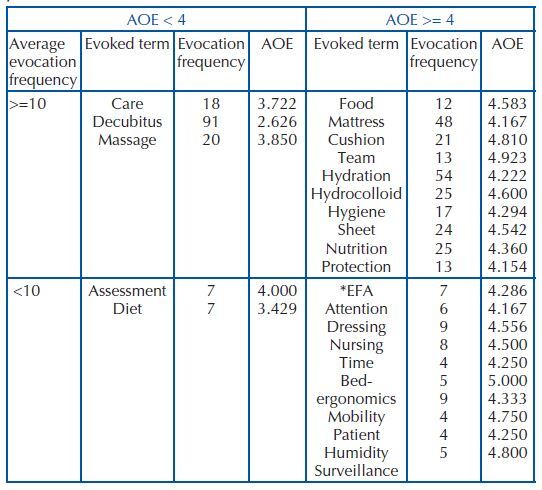
Four-quadrant model of the inducing term “pressure injury preventive measures” – Porto Velho, RO, Brazil, 2019.

In the survey on screen, the cut-off point for the minimum evocation frequency was 4, excluding terms below this frequency and the average frequency of 10. From these frequencies, the program calculated the Average Order of Evocations (AOE), which corresponded to 3.8, being adjusted to 4. The four-quadrant model is shown in Chart 1.

*EFA – essential fatty acid.

Among the terms evoked about the preventive measures to PI present in the ULQ, there is “decubitus”, an element most frequently, readily evoked and of relevance to the group. This term refers to a dimension of the image related to such an object, which is patients positioned in the hospital bed to relieve the pressure of a part of the body.

The element “care” was the third most evoked word, presenting the second smallest AOE. Its presence in the central nucleus may be revealing both a practical dimension, of implementing an action that promotes PI prevention, and the (symbolic) understanding that preventive measures are care that gives meaning to the nursing performance towards patients as one of its responsibilities.

This understanding of preventive measures as nursing care as a group consensus guides positions and is prescriptive of behaviors. On the other hand, the non-performance of this care by the nursing team goes against this social norm.

The term “massage” occupied the second place among the most evoked elements, being the third most readily evoked word by the group. Therefore, it expresses a relevant cognition in relation to the object and, thus, suggests that it is preventive care for PI.

The elements that made up the URQ are those that support the meaning present in the central core. They are evoked cognems with high frequency, but belatedly evoked. The terms “food”, “mattress”, “cushion”, “team”, “hydration”, “hydrocolloid”, “hygiene”, “sheet”, “nutrition” and “protection” were characterized as support to the practical dimension of care, since they are associated with care actions.

The evocations “mattress”, “cushion”, “sheet” and “hydrocolloid” may be showing that, in order to provide care for PI prevention, especially the change in position, attention is needed to the technologies used to support this care, i.e., the choice and installation of a suitable mattress for the redistribution of pressure between the parts of the body; use of cushions to maintain patient position; and the correct arrangement of the sheet, avoiding folds that can generate friction and harm skin integrity^(7)^.

The evocation “team”, present in this same quadrant, combined with “hydration”, “nutrition”, “food” and “protection”, is indicative of the recognition by nursing professionals that care for PI prevention also depends on the interdisciplinary team, which contributes to the prescription of measures for skin integrity.

In the contrast zone, assessment and diet elements appeared. It is important to point out that this zone presents terms with low frequency and average of evocation, but considered important by the subjects, which complement or reinforce the notion of the first periphery or indicate a different representation of a minority group. From this perspective, the terms “assessment” and “diet”, present in this LLQ, complement the notion of interdisciplinarity of the first periphery. The assessment refers to the use of patient assessment instruments regarding the risk of developing PI and the implementation of multidisciplinary interventions aimed at prevention based on this classification. The term “diet” indicates good food intake in preventing PI occurrence, which requires nutritional assessment.

In the second periphery (LRQ) are the terms “EFA”, “attention”, “dressing”, “nursing”, “schedule”, “bed-ergonomics”, “mobility”, “patient”, “humidity” and “surveillance”. These terms bring nursing attributions in relation to patient care in PI prevention. The articulated terms “nursing” and “patient” express these care activities, which include dressing or using EFA to protect/recover the skin, pay attention to the time to mobilize patients, and monitor patient humidity.

In the double negation test, presented in Table 1, the evocations “care” and “decubitus” had their centrality confirmed, as they reached a rebuttal percentage of 97.5%, unlike “massage”, which was not non-negotiable for the group.

**Table 1.** Double denial test of the central core of social representations on preventive measures against pressure injuries – Porto Velho, RO, Brazil, 2019.

Element No, I cannot 95% CI* n %	
Massage Decubitus Care	28 (70%) (53.0 to 83.4) 39 (97.5%) (86.4 to 99.9) 39 (97.5%) (86.4 to 99.9)

*CI - confidence interval.

**Figure 1.**
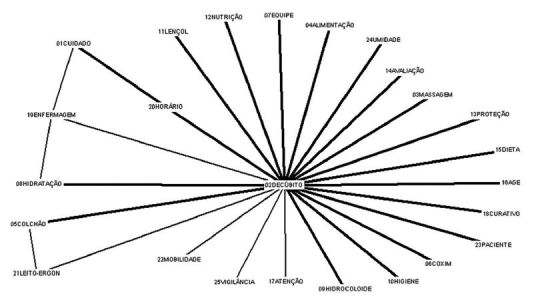
Tree of similarity of nursing professionals’ evocations. Porto Velho, RO, Brazil, 2019.

The results of the similarity analysis expressed in Figure 1 showed, at the central level of the tree, the term “decubitus” being the cognition that most established connections. Furthermore, the meanings attributed by the group articulated the words “decubitus”, “time”, “care” and “nursing”, “decubitus”, “nursing” and “hydration”, “decubitus”, “mattress” and “bed-ergonomics”. The lexicon “massage” did not confirm its centrality, being a peripheral element.

**Caption:** 1 – care; 2 – decubitus; 3 – massage; 4 – food; 5 – mattress; 6 – cushion; 7 – team; 8 – hydration; 9 – hydrocolloid; 10 – hygiene; 11 – sheet; 12- nutrition; 13 – protection; 14 – assessment; 15 – diet; 16 – EFA; 17 – attention; 18 – dressing; 19 – nursing; 20 – time; 21- bed-ergonomics; 22 – mobility; 23 – patient; 24 – humidity; 25 – surveillance.

## DISCUSSION

Data on the constitutive elements of SR on PI preventive measures of nursing teams indicated that the central nucleus was formed by the words “decubitus” and “care”. Therefore, it was evidenced that implementing PI preventive measures is a nursing care that gains concreteness and materializes in the action of changing patient position (decubitus). This meaning referred to the normative, practice and image dimensions, which are part of the SR structure.

The structural approach points out that SR are made up of two dimensions, structural, relating to information, beliefs, attitudes and opinions in relation to the represented object, and organizational, which reveals how such contents establish connections with each other. In this structural approach, SR consider that the central nucleus has two essential characteristics: one functional and one normative^(15)^.

The normative and functional aspects were established in the model of the SR theorist by Jean-Claude Abric. The normative dimension is the expression of judgments, stereotypes and opinions about objects. Thus, in situations where affective, social or ideological issues are involved in the object, the normative dimension is present in the central nucleus, expressing the group’s consensus. From this perspective, this dimension expresses the subjects’ attitudes, from which information is assessed and valued in SR. In the functional dimension, there are the elements that represent actions to be performed on or in the object^(15)^.

The imagery dimension is also important to be identified, as the images give concreteness to the object and, with this, they become naturalized and start to be used as a reference grid for understanding the phenomenon^(13)^.

This theoretical interpretation of the dimensions that structure the SR of preventive measures can be contrasted with the scientific evidence, produced from the results of national and international studies on the subject. Such studies indicate contradictions between the discourse and practice of PI prevention as well as a low compliance by professionals with PI prevention practices^(8–10)^.

In one of the studies, the PI prevention practices of 422 nurses in public hospitals in Ethiopia were assessed. Data from the questionnaire showed that 51.9% of nurses reported good practices in PI prevention, however, in the observation, it was found that 45.3% practiced activities categorized as adequate for PI prevention^(8)^.

Another survey also developed in Ethiopia between 2017-2018 assessed PI prevention practices among 125 nurses from public hospitals. Adequate knowledge and good practice were considered when nurses reached 80% or more of correct answers about PI prevention and good practices, respectively. Of the participants, 29.5% had inadequate knowledge, while in relation to practices, 82.2% had a poor practice of PI prevention. Still, 50.3% always implemented PI prevention practices, 36.4% did sometimes and 15% never did^(9)^.

A national investigation assessed the omission of nursing care based on the perception of 267 nursing professionals operating in hospitalization units of a teaching hospital. Omitted care was considered those whose answers of participants to the questionnaire included “never”, “rarely” and “occasionally” performed. Regarding the prevalence of omissions, the main ones were sitting the patient out of bed (70.3%) and ambulation three times a day (69.1%). Changing the decubitus patient every two hours had an omission of 29.7%, being higher among nurses (43.6 versus 23.9%) compared to other nursing professionals^(11)^.

This contrast with the supporting literature, which indicates omissions and a low rate of good PI prevention practices, allows us to consider that the term “decubitus”, established as a central element of SR, it can mean a politically correct discourse on the phenomenon, which masks negative assessments related to its implementation. This is because not implementing the change of decubitus is counternormative, i.e., it goes against the social norm of nursing responsibility care.

Thus, the hypothesis is raised that the non-implementation of preventive measures, in particular, the change of decubitus, may be an element of the mute zone of SR. The mute zone represents spaces of representations that, although shared by the group, are not always revealed in the speeches, as they go against the prevailing social norms^(17)^. Thus, the first words evoked may be those that are socially acceptable in a given context; therefore, a social desirability effect that masks negative assessments.

The term “massage” was not confirmed as an element of the central core, but the fact that it was promptly evoked demonstrates that it remains relevant and salient for the group investigated in the social construction of the phenomenon, despite no longer being considered a preventive measure in evidence-based protocols and guidelines since 2013^(1,7)^.

It is emphasized that the constitution of the central nucleus may be directly linked to historical issues. In this regard, it is pointed out that individual memory is subject to collective memory; therefore, it is understood that the spaces of memories are social and are determined to be built and reconstructed with elements of the past, based on social frameworks^(18)^.

In the case of PI preventive measures, such as guidelines recommended by the scientific community in the area at a given time, they become a consensus. As a result, they circulate and spread among the professionals who are involved with them, taking root in their daily practice. When talking about this phenomenon, they reinterpret it and, due to the relevance that some aspects assume in this symbolic construction, they come to the fore, as was the massage. Moreover, 79% of participants reported lack of access to current prevention guidelines.

Some investigations reinforce this statement, when they demonstrate that massage is still incorporated into the practice of PI prevention as a care implemented in nursing care^(19,20)^. One of them was carried out in a university hospital with 38 nurses, to identify the knowledge of care nurses regarding PI prevention and care in medical and surgical clinic units. Massage in bony prominences was one of the items of lesser accuracy, plus items related to the use of devices, such as water glove, pads and in relation to positioning^(20)^.

Regarding the peripheral core of SR, it allows SR to be anchored in reality. Thus, it brings elements that determine and organize the behavior related to the object, the practices to be carried out^(14)^. In addition to the function of implementing the central system, it has the function of regulating and adapting that system to the real situation’s constraints and characteristics. In this regard, it is flexible to the immediate context, allowing the integration of subjects’ individual experiences in relation to the phenomenon into the SR^(14)^.

In the case of the research, the terms evoked mean that care, through changing position, in itself, is not enough to prevent PI, and it is necessary to think about the other elements that support this preventive nursing care, such as technologies (mattress, cushion and sheet) and fundamental care (hygiene, food, hydration). This understanding is corroborated by the national recommendations for PI prevention^(7)^ and also by the results of investigations that describe interventions implemented by nursing professionals.

In one of these studies developed in the ICU, the findings pointed to the most frequent interventions: assessment of patients’ activity-mobility; physical examination on admission; keeping the skin hydrated; decubitus alternation; body hygiene; and use of pyramidal mattress^(21)^.

Although participants have represented preventive measures as nursing care, the participation of other professionals in the interdisciplinary team, raised by the terms evoked in the first periphery and contrast zone, seems to be a sensitive point, with the possibility of difficulties with cooperative work and failures in the use of preventive measures. Thus, the question arises whether in fact this multidisciplinary assessment, mainly based on the use of assessment instruments, is consistently incorporated into professional practice.

This question is based on research on the subject, because when they address skin integrity assessment and the risk of developing PI, they reinforce that it is a complicating factor for comprehensive care that involves preventive measures^(22,23)^.

This analysis is illustrated by the study carried out with 97 nurses and nursing assistants in Colombia, to assess compliance with nursing care for PI prevention, in which 45% of actions were categorized as non-compliance, and 35% as excellent compliance. The actions to prevent greater compliance were to execute a care plan (54%), use the elements available for prevention (54%) and to record risk factors (53%). The actions least employed by nurses were to assess the degree of risk with the use of scales and reassess according to patients’ status (58%)^(22)^.

Researchers assessed PI prevention practices among tertiary hospital nurses in India and developed guidelines for pressure point care. According to their findings, 93.3% of nurses in the sample of 157 participants were performing adequate practices, but they highlighted that inadequate practices still persist, such as the lack of a risk assessment tool for PI, i.e., nurses did not assess systematically the skin for the risk of the presence of PI^(23)^.

Finally, in the second periphery, the term “bed-ergonomics” stands out, which portrays a concern with professionals’ health, by adjusting the appropriate height of the bed to the position of their body, with a view that they do not have musculoskeletal problems and promote appropriate preventive care. It is conjectured that the term “bed-ergonomics” may indicate an affective dimension of SR.

Affections encompass the feelings and emotions that are impregnated in human existence. Feelings include moods (anxiety, depression) and assessments (positive/negative), while emotions involve fear/anger. The expression of thought in prescriptions, judgments, concepts is not indifferent to the other, but is permeated by these affections involved in the relationship with the world^(24)^.

Therefore, it is considered that negative affects can be mobilized by the action of changing position, since it can refer to the assessment of the physical repercussions caused in professionals from the use of preventive measures in patients. Therefore, it may have repercussions on the practical dimension of professionals with lower compliance with preventive measures.

This non-access to preventive measures or their implementation incorrectly or discontinued if it constitutes an error that can result in harm to patients. Therefore, the SR structure brings, depending on the activated psychosocial elements, implications for patient safety, with a greater risk of developing PI as an adverse event. By understanding the psychosocial logic of this phenomenon, the results bring contributions to nursing, as they make it possible to think about interventions that constitute barriers to the recurrence of errors and promote patient safety.

The limitations of this research included the impossibility of applying WAT with all participants at the same time, in order to ensure greater reliability to the technique.

## CONCLUSION

The SR structure had as its central core the evocations “care” and “decubitus”. The imagery dimension of change in position brought concreteness to the phenomenon, from which there is a hierarchy of preventive measures. The term “care” referred to the normative dimension of SR, in the understanding that the use of preventive measures is a care that is the responsibility of nursing, and practice, based on preventive care actions using technologies such as mattresses, cushions, sheets and hydrocolloids. The peripheral evocation bed-ergonomics was associated with a negative affective dimension, which may imply the non-compliance of professionals with preventive practices.

These results serve as a basis for educational proposals in the scenario studied on PI prevention with a focus on the reorganization of symbolic, imagery and affective elements that organize/structure the meanings and practices of nursing professionals about this phenomenon, with a view to greater compliance with preventive measures and patient safety.
